# Night-time splinting after fasciectomy or dermo-fasciectomy for Dupuytren's contracture: a pragmatic, multi-centre, randomised controlled trial

**DOI:** 10.1186/1471-2474-12-136

**Published:** 2011-06-21

**Authors:** Christina Jerosch-Herold, Lee Shepstone, Adrian J Chojnowski, Debbie Larson, Elisabeth Barrett, Susan P Vaughan

**Affiliations:** 1 Faculty of Medicine and Health Sciences, University of East Anglia, Norwich NR4 7TJ, UK; 2 Department of Orthopaedics and Trauma, Norfolk and Norwich University Hospitals NHS Foundation Trust, Norwich NR4 7UY, UK; 3 Department of Occupational Therapy, Norfolk and Norwich University Hospitals NHS Foundation Trust, Norwich NR4 7UY, UK

## Abstract

**Background:**

Dupuytren's disease is a progressive fibroproliferative disorder which can result in fixed flexion contractures of digits and impaired hand function. Standard treatment involves surgical release or excision followed by post-operative hand therapy and splinting, however the evidence supporting night splinting is of low quality and equivocal.

**Methods:**

A multi-centre, pragmatic, open, randomised controlled trial was conducted to evaluate the effect of night splinting on self-reported function, finger extension and satisfaction in patients undergoing fasciectomy or dermofasciectomy. 154 patients from 5 regional hospitals were randomised after surgery to receive hand therapy only (n = 77) or hand therapy with night-splinting (n = 77). Primary outcome was self-reported function using the Disabilities of the Arm, Shoulder and Hand (DASH) questionnaire. Secondary outcomes were finger range of motion and patient satisfaction. Primary analysis was by intention to treat.

**Results:**

148 (96%) patients completed follow-up at 12 months. No statistically significant differences were observed on the DASH questionnaire (0-100 scale: adjusted mean diff. 0.66, 95%CI - 2.79 to 4.11, p = 0.703), total extension deficit of operated digits (degrees: adjusted mean diff 5.11, 95%CI -2.33 to 12.55, p = 0.172) or patient satisfaction (0-10 numerical rating scale: adjusted mean diff -0.35, 95%CI -1.04 to 0.34, p = 0.315) at 1 year post surgery. Similarly, in a secondary per protocol analysis no statistically significant differences were observed between the groups in any of the outcomes.

**Conclusions:**

No differences were observed in self-reported upper limb disability or active range of motion between a group of patients who were all routinely splinted after surgery and a group of patients receiving hand therapy and only splinted if and when contractures occurred. Given the added expense of therapists' time, thermoplastic materials and the potential inconvenience to patients having to wear a device, the routine addition of night-time splinting for all patients after fasciectomy or dermofasciectomy is not recommended except where extension deficits reoccur.

**Trial registration:**

The trial was registered as an International Standard Randomised Controlled Trial ISRCTN57079614

## Background

Dupuytren's disease (DD) is a disabling hand condition that is thought to affect more than 2 million people in the UK [1]. Standard surgical treatment includes division of the cords (fasciotomy) or excision (partial or total fasciectomy) [1]. Long term recurrence may be reduced by excision of cords/nodules with the overlying skin and full thickness skin grafting (dermofasciectomy) [2].

Less invasive percutaneous needle fasciotomy [3] and injectable collagenase ('chemical knife') [4] are alternative outpatient delivered procedures resulting in good short-term contracture release, however fasciotomy is also associated with a much higher recurrence rate compared to limited fasciectomy [5].

Surgical excision (fasciectomy) of the diseased cords continues to be a widely used treatment option aimed at correcting the digital contractures and to improve hand function. Rehabilitation post surgery by hand therapists is recommended [6] to control scar formation, prevent secondary complications, and to restore movement and hand function. The use of thermoplastic extension splints worn at night and/or daytime is advocated by many as part of the post-operative rehabilitation [6,7]. However a systematic review [8] concluded that there is conflicting evidence on the effectiveness of post-operative splinting for Dupuytren's disease with both positive and negative results reported.

The paucity of research to date is reflected by the inconsistent use of splinting in clinical practice. A survey of 573 orthopaedic consultants in the UK showed that 33% used splinting most or all of the time [9] yet another survey of 141 surgeons found that 84% advocated night splinting [10].

Finally, splints may be inconvenient for patients to wear and the materials, therapists' time and skill required to fabricate these custom-made devices are an added expense to health care providers.

The aim of this study was to compare the effect of post-operative static night splinting in addition to hand therapy with hand therapy only on patient reported upper extremity symptoms and disability, composite active digital range of motion, patient satisfaction and recurrence of contracture at one year.

## Methods

### Patients and Setting

We conducted an open, pragmatic, multi-centre, randomised controlled trial of night-time static splinting worn for 6 months after contracture release to assess the effect on self-reported upper extremity function, active range of movement in all digits and patient satisfaction at 12 months follow-up.

Between October 2007 and January 2009 patients referred with Dupuytren's disease affecting one or more digits of either hand and requiring fasciectomy or dermofasciectomy were invited to participate. Patients had to be over 18 years of age and competent to give fully informed written consent. Patients presenting with contracture of the thumb or first web-space only were excluded.

Identifying eligible patients was conducted in five National Health Service (NHS) Hospital Trusts in East Anglia involving a total of 16 operating surgeons. The surgery and all post-operative interventions were delivered within these five secondary care centres. Data collection at baseline and all follow-up assessments for the trial were undertaken in the patients' own homes by two trained research associates.

The protocol was approved by the Cambridgeshire 2 Research Ethics Committee (REC 07/Q0108/120) in July 2007 and the Research Governance and Ethics Committee of each participating hospital. All patients gave fully informed written consent.

### Randomisation

Patients were randomised after surgical release at their first post-operative appointment by the treating hand therapist (within 2 weeks after surgery and following removal of sutures). Randomisation was stratified by centre (five centres) and by surgical procedure (fasciectomy or dermofasciectomy) in block lengths of 4. The allocation sequence was generated and administered independently through a central telephone randomisation service at the Clinical Trials Unit, University of East Anglia. Neither the treating therapists nor the patients were blinded to treatment allocation.

### Interventions

The intervention being studied in this trial was static splinting worn at night-time for 6 months after surgical release in addition to usual hand therapy. As this was a pragmatic trial we did not attempt to standardise surgical procedure. Surgeons were allowed to use their preferred surgical techniques tailored to the severity and extent of the contracture. Hand therapy could not be standardised as it is a complex intervention using multiple modalities to treat a wide range of post-operative problems and has to be tailored to the patient's needs. In order to collect data on the range of modalities and treatments used by hand therapists, such as oedema control, exercises or advice, hand therapists were asked to complete a treatment reporting form at each session. This was also used to record post-operative complications and reasons for treatment deviation from protocol.

#### Splint group

Patients randomised to the splint group were provided with a custom-made thermoplastic splint fabricated by the treating hand therapist. The splint was designed to accommodate the operated rays of the hand with the metacarpophalangeal joint (MCPJ) and/or proximal interphalangeal joint (PIPJ) held in maximum extension without causing any tension to the wound. After a further 3 weeks when the wound has healed and scar tissue begins to mature the splint could be remoulded to achieve a greater extension force designed to prevent any loss of extension from surgical scar contracting.

Patients were instructed to wear the splint at night only and were given a splint diary in which they recorded weekly how many nights out of 7 they had actually worn the splint and any reasons why the splint was not worn.

#### No splint group

The experimental treatment involved providing usual hand therapy only. However, it was deemed unethical to withhold the application of a splint in patients who developed contractures and which did not respond to hand therapy only. Clinical staff from all five centres were consulted prior to the trial to devise criteria for 'per protocol' deviations for patients allocated to the no-splint group in the event that they experience a rapid and substantial loss of finger extension. At the first hand therapy visit and following randomisation active range of movement of MCPJ and PIPJ was measured by the hand therapist with a goniometer and recorded. At the 2^nd ^visit (normally 1 week later) this range of motion was re-measured and if the patient had a net loss of 15° or more at the PIPJ and/or a net loss of 20° or more at the MCPJ of the operated fingers, they were then given a splint and splint diary. The dates and reasons for these protocol deviations were recorded by the hand therapists and the trial coordinator was notified. Any patients allocated to the no-splint group who were given a splint for any other reasons such as surgeon request were recorded as protocol violations.

### Outcome assessment

The primary outcome measure was self-reported upper extremity function using the 30-item Disabilities of the Arm, Shoulder and Hand (DASH) questionnaire. The DASH is a validated measure of symptoms and physical function for patients with a wide range of musculoskeletal disorders of the upper extremity and has been extensively used in reporting the outcome of surgery for DD before [11-13]. The questionnaire was mailed to patients for self-completion prior to the research associates' visit at which secondary assessments were taken.

Secondary outcomes were active range of motion of the MCPJ, PIPJ and distal interphalangeal joint (DIPJ) of operated digits and patient satisfaction. Range of motion was assessed with a Rolyan (Sammons Preston, USA) finger goniometer and following a standardised protocol [14]. Training was given prior to the trial and both research associates undertook comparative assessments at regular intervals. Individual joint movement of full active flexion and extension was summed for each operated finger and averaged to give a total active extension (TAE) and total active flexion (TAF). Any hyperextension of a joint was recorded but converted to zero for the analysis to prevent underestimation of extension deficit.

Primary and secondary outcomes were assessed prior to surgery, and at 3, 6 and 12 months after surgery. Patient satisfaction was assessed only at 6 and 12 months by asking the patient to rate their satisfaction with the outcome using an 11-point verbal rating scale where 0 indicates complete dissatisfaction and 10 complete satisfaction. The original protocol also indicated recurrence at 1 year as a secondary outcome, however this was abandoned due to the inherent difficulties of distinguishing a true recurrence from a residual scar contracture, the lack of agreed definition of recurrence [15] and the subjectivity in visually inspecting and palpating the hand.

### Statistical approaches

#### Sample size

Using the DASH as the primary outcome measure, where a difference of 15 points is considered to be a minimal important change (MIC) [16] and using a between-group standard deviation of 22 points [17] a total of 51 patients would be needed in each group for a power of 90%. Allowing for a 20% loss to follow-up a total of 128 randomised patients was aimed for.

#### Analysis methods

Baseline demographic and clinical characteristics were compared between groups using descriptive statistics to assess any potential disparities between groups.

The primary outcome, the DASH score, was found to be very right-skewed and therefore a logarithmic transformation was applied to produce a more symmetric distribution, assumed to be Normal. A general linear model was used to formally analyze the between group mean difference adjusting for the baseline DASH score, recruiting hospital and type of surgery (the latter two variables were used to stratify randomisation).

Range of motion, that is total active flexion (TAF) and total active extension (TAE) both appeared to follow a Normal distribution. These were also analysed using a general linear model with adjustment for baseline TAF or TAE values, recruiting hospital and type of surgery. Similarly, at 6 and 12 months, the patient satisfaction score was analysed using a general linear model with adjustment for recruiting hospital and type of surgery.

A Poisson model was used to analyse number of therapy sessions with adjustment for recruiting hospital and surgery type. Similarly, a Logistic regression model was used to analyse the use of a dynamic splint again with adjustment for recruiting hospital and surgery type.

As appropriate to pragmatic trials an intention-to-treat approach was used as the primary analysis strategy in which all patients were analysed according to their initial group allocation. However, no data imputation was used for missing follow-up data as these were very few.

A secondary per-protocol analysis was conducted comprising those individuals that adhered to the treatment protocol. In the splint group, adherence was defined *a priori *as wearing the splint for at least 50% of nights in the first three months after surgery. In the no-splint group the per protocol analysis included those individuals who did not use a splint or were managed as 'per protocol' and given a splint once their extension loss exceeded the criterion agreed by the trial management group.

For the intention-to-treat analysis the statistician (LS) was sub-group blind.

The trial was registered as an International Standard Randomised Controlled Trial, number ISRCTN57079614 on http://www.controlled-trials.com. The trial was funded by Action Medical Research Charity, UK.

## Results

Between October 2007 and January 2009 218 patients were referred as eligible and invited to participate. 46 patients declined, one patient withdrew and one patient died. A further 16 patients were excluded because either their surgery had been cancelled or postponed (n = 8), they were mistakenly referred after surgery (n = 6), one patient could not be contacted and one patient was not referred to hand therapy (n = 1).

**Figure 1:**
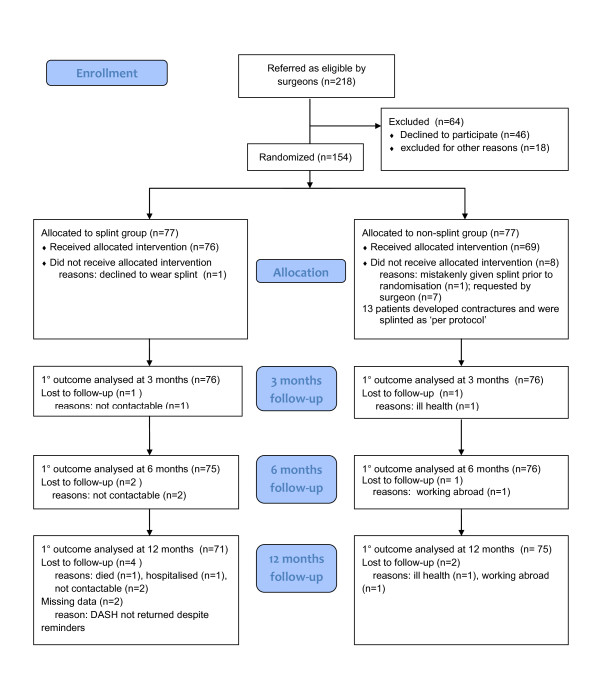
**CONSORT flowchart for SCoRD trial**.

A total of 154 patients were thus enrolled into the trial from December 2007 to January 2009 and randomly allocated to either trial arm (see Figure 1). Follow-up continued until January 2010 and six patients were lost to follow-up (4%). Of the 77 patients allocated to receive a splint, one patient refused the splint and was also lost to follow-up. In the no-splint group 69 of the 77 patients received the allocated intervention of hand therapy only. There were eight protocol violations in the no-splint group: one patient was given a splint by mistake prior to randomisation and seven patients were given a splint immediately at their first hand therapy appointment either due to surgeon request or because the patient already presented with a loss of extension. 13 patients allocated to the no-splint group (17%) went on to develop a contracture of the PIPJ which exceeded the agreed threshold and were subsequently given a splint as per protocol.

The baseline demographic and clinical characteristics for the two groups are given in Table 1. Both groups were similar with regards to clinical characteristics except for the proportion of patients who had one or more digits operated with a slightly larger proportion of multiple digit involvement in the no-splint group. A fasciectomy was the most common procedure with only 16 patients receiving a dermofasciectomy.

**Table 1:** Baseline demographic and clinical characteristics

		*Splint group*	*No splint group*
		*N = 77*	*N = 77*
Centre	Cambridge	13 (17%)	13 (17%)
	Ipswich	17 (22%)	18 (23%)
	Norwich	30 (39%)	29 (38%)
	Peterborough	5 (6%)	4 (5%)
	West Suffolk	12 (16%)	13 (17%)
			
Age	Mean (SD)	67.2 (10.0)	67.5 (9.2)
			
Sex	Ratio male: female	61:16	59:18
			
Occupation	Working	24 (31%)	29 (38%)
	Seeking Work	1 (1%)	0
	Retired/Not working	52 (68%)	48 (62%)
			
Surgery Type	Dermofasciectomy	7 (9%)	9 (12%)
	Fasciectomy	70 (91%)	68 (88%)
			
Operated Digit	Index	5 (7%)	3 (4%)
	Long	7 (9%)	16 (21%)
	Ring	37 (49%)	26 (34%)
	Small	56 (74%)	53 (69%)
			
No operated rays	One	50 (66%)	60 (78%)
	Two	23 (30%)	13(17%)
	Three	3 (4%)	4 (5%)
			
Previous Surgery	Yes	11 (14%)	12 (16%)
	No	66 (86%)	65 (84%)
			
Woodruff Grade	2 (MCP only)	3 (4%)	7 (9%)
	3 (MCP and PIP, single digit)	42 (55%)	45 (58%)
	4 (as 3 multiple digits)	32 (41%)	25 (33%)
			
Initial DASH Score	Mean (SD)	16.4 (15.1)	15.4 (13.2)
			
Initial TAF*	Mean (SD)	223.8 (20.9)	226.2 (15.0)
Initial TAE*	Mean (SD)	50.7 (22.2)	51.1 (18.8)

TAF = total active flexion of MCPJ, PIPJ and DIPJ of operated digit(s) only

TAE = total active extension of MCPJ, PIP and DIPJ of operated digit(s) only

Table 2 presents the results of the ITT analysis for primary and secondary outcomes at 3, 6 and 12 month by treatment group and the adjusted mean difference with 95% confidence interval. There were no statistically significant differences at 12 months between the two groups in DASH score (0.66, -2.79 to 4.11, p = 0.703), degrees of total active flexion of operated digits (-2.02, -7.89 to 3.85, p = 0.493), degrees of total active extension deficit of operated digits (5.11, 2.33 to -12.55, p = 0.172). Both groups were satisfied with the outcome at 12 months (mean 8.5 splint group and 8.9, no-splint group) and did not differ significantly (-0.35, -1.04 to 0.34, p = 0.315). Similarly, no significant differences were found at 3 or 6 months.

**Table 2:** Intention-to-treat analysis for primary and secondary outcomes (mean and standard deviation)

		*Splint group*	*No splint group*	*Adjusted Difference 95% C.I*.	*p-value*
3-month	DASH (0-100)	9.6 (12.8)	10.8 (12.5)	-1.48	0.403
		n = 76	n = 76	(-5.02 to 2.06)	
	LogDASH	1.74 (1.17)	1.87 (1.18)	-0.16	0.372
		n = 76	n = 76	(-0.50 to 0.19)	
	TAF (degrees)	213.0 (26.5)	217.6 (22.5)	-3.49	0.326
		n = 75	n = 76	(10.57 to -3.59)	
	TAE (degrees)	-32.9 (19.6)	-30.9 (20.7)	3.30	0.209
		n = 75	n = 76	(1.93 to -8.53)	
					
6-month	DASH	7.9 (11.4)	7.1 (10.7)	0.20	0.890
		n = 75	n = 76	(-2.72 to 3.12)	
	LogDASH	1.47 (1.21)	1.38 (1.21)	0.07	0.704
		n = 75	n = 76	(-0.29 to 0.43)	
	TAF	220.6 (25.2)	225.8 (21.6)	-4.16	0.199
		n = 74	n = 76	(-10.6 to 2.29)	
	TAE	-31.0 (23.3)	-28.4 (21.1)	4.52	0.142
		n = 74	n = 76	(-1.61 to 10.65)	
	Patient Satisfaction (0-10)	8.7 (1.89)	9.0 (1.23)	-0.28	0.286
		n = 75	n = 76	(-0.81 to 0.24)	
					
12-month	DASH	7.0 (14.6)	6.0 (9.2)	0.66	0.703
		n = 71	n = 75	(-2.79 to 4.11)	
	LogDASH	1.24 (1.21)	1.23 (1.20)	-0.02	0.914
		n = 71	n = 75	(-0.38 to 0.34)	
	TAF	223.8 (25.7)	227.3 (19.5)	-2.02	0.493
		n = 72	n = 75	(-7.89 to 3.85)	
	TAE	-32.9 (27.4)	-29.6 (23.3)	5.11	0.172
		n = 72	n = 75	(-2.33 to 12.55)	
	Patient Satisfaction	8.5 (2.33)	8.9 (1.79)	-0.35	0.315
		n = 73	n = 75	(-1.04 to 0.34)	

The mean number of therapy sessions was 5.1 (SD 2.5) in the splint group and 5.6 (SD 3.5) in the no -splint group. There were no significant differences in number of therapy sessions between the two groups (adjusted odds ratio: 0.93, 0.81 to 1.07, p = 0.305). The use of subsequent dynamic daytime splints for residual PIPJ contractures was similar for both groups (13 patients in splint group and 14 in no-splint group) and was not statistically significant (adjusted odds ratio: 0.91, 0.35 to 2.36, p = 0.839).

A secondary per protocol analysis was conducted on all those patients in whom the treatment protocol was adhered to. This included those patients allocated to the splint group and wore the splint for at least 50% of nights in the first 3 months. In those allocated to the no-splint it includes the 13 patients given a splint due to ensuring contracture. Table 3 gives the results for the per protocol analysis for primary and secondary outcomes at 3 months, 6 months and 12 months. No statistically significant differences were found on any of the outcomes and at any time points.

**Table 3:** Per protocol analysis for primary and secondary outcomes at all timepoints

		*Splint group*	*No splint group*	*Adjusted Difference 95% C.I*.	*p-value*
		n = 65	n = 68		
3-month	DASH (0-100)	9.8 (13.5)	10.7 (13.0)	-2.19	0.270
				(-6.15 to 1.77)	
	LogDASH	1.72 (1.20)	1.84 (1.20)	-0.20	0.302
				(-0.58 to 0.18)	
	TAF (degrees)	211.9 (26.5)	218.7 (21.5)	-4.51	0.245
				(-12.2 to 3.2)	
	TAE (degrees)	33.0 (19.4)	31.1 (21.0)	3.49	0.210
				(-2.06 to 9.04)	
					
		n = 64	n = 68		
6-month	DASH	8.4 (12.1)	7.4 (11.0)	-0.13	0.936
				(-3.35 to 3.09)	
	LogDASH	1.51 (1.24)	1.38 (1.24)	0.05	0.793
				(-0.34 to 0.44)	
	TAF	220.4 (23.0)	226.5 (21.7)	-3.92	0.242
				(-10.59 to 2.75)	
	TAE	30.9 (23.5)	29.0 (21.9)	4.38	0.188
				(-2.23 to 10.99)	
	Patient Satisfaction (0-10)	8.65 (2.00)	9.00 (1.23)	-0.34	0.254
				(-0.92 to 0.24)	
		n = 62‡	n = 67		
12-month	DASH	7.4 (15.5)	6.1 (9.6)	0.44	0.821
				(-3.41 to 4.29)	
	LogDASH	1.27 (1.22)	1.20 (1.23)	0.02	0.919
				(-0.37 to 0.41)	
	TAF	223.4 (23.4)	228.0 (19.3)	-1.87	0.527
				(-7.78 to 4.04)	
	TAE	32.6 (27.7)	30.1 (24.4)	5.12	0.211
				(-3.01 to 13.3)	
	Patient Satisfaction	8.68 (2.17)	8.85 (1.84)	-0.18	0.622
				(-0.90 to 0.54)	

‡ for DASH and log DASH splint group n = 61

Mean adherence to splint wear in the first 3 months was 74.6% of nights (SD = 29.4%) and only 12 patients did not meet the adherence criterion (splint worn ≥50% of night for first 3 months). Reasons for non-adherence or discontinuing splint wear were documented in the splint diary. The most commonly cited reason (n = 12) was the patient no longer perceiving any benefit from wearing the splint, other reasons were: causing hand stiffness, causing discomfort or pain, sleep disturbance, advised to discontinue by surgeon, ill fitting or causing a rash.

## Discussion

This pragmatic trial has shown that the policy of using routine night-time static splints in addition to usual post-operative hand therapy does not offer any additional benefit in terms of self-reported hand function and disability, active composite range of flexion or extension or patient satisfaction. Both groups improved over the 12 months follow-up period in their DASH score. However any between group differences were not statistically significant and the 95% confidence intervals included values of 5 points or less which are well below the threshold for a minimal important change in DASH.

As surgery is primarily aimed at releasing contracted tissues and splints designed to maintain this extension, total extension deficit is an important clinical measure of change and a more proximate outcome. However the ITT analysis found no statistically significant differences between the two groups on either active extension or flexion.

A potential weakness of the trial is that the primary outcome measure was patient-reported and participants could not be blinded. Secondary outcomes were collected by the research associates who were also not blinded, although they were independent of the clinical staff delivering the interventions. The lack of blinding could introduce a bias but this bias is more likely to be in favour of splinting (i.e. those patients with an active intervention more likely to report favourable results). The fact that the trial did not find evidence in support of splinting would mitigate against the possible assessment bias from the patients' or researchers' expectations to have a better outcome from splinting.

Our trial exceeded the required sample size for 90% power, had a very high follow-up rate (96%) and even in the secondary per protocol analysis the sample size still exceeds 51 in each group, therefore the possibility of a Type II error remains low, and yet the confidence intervals exclude the minimally important difference for the DASH.

There are several possible explanations for our results. The primary outcome measure is a well-validated outcome measure for upper limb function and disability. However, DASH is a region-specific upper extremity measure and not disease-specific. It may therefore lack sensitivity to change in patients with Dupuytren's contracture. A clinically important change of 15 points as a criterion is a large effect size especially when considering that the baseline mean score prior to surgery was only 16 points, thus indicating minimal disability. Range of movement of digital joints could be argued as a more proximate and objective indicator of contracture severity yet the differences between groups were also non-significant with small and narrow confidence intervals confirming that a splint does not offer any additional benefit in terms of active extension or flexion of affected digits.

A second possible reason for a lack of difference could be dilution bias especially through non-adherence in the splint group. Patients were asked to complete a weekly splint diary, however overall mean adherence with splint wear was high and only 12 patients in the splint group were deemed as non-adherent (15.6%) at 3 months. The results of the per protocol analysis which includes only those who met the adherence criterion of splint wear for ≥50% differences were still non-significant for all primary and secondary outcomes indicating that even when the splint is worn or adhered to a lack of effect is still evident. One weakness is that these adherence rates relied on patient completed diaries. Whilst the diaries were collected by research associates (not the treating therapists or surgeon) and therefore encouraged patients to be honest, independent verification of actual splint wear was not possible.

A third plausible reason for the lack of effect could be that the amount of tension provided through a static night splint is not sufficient to remodel scar tissue. In a study investigating full time casting in patients with an orthopaedic injury improvement in PIP flexion contracture was related to the total time that the cast was worn with greater extension achieved the longer the cast was worn [18]. However it is unknown if the same principle applies to an intermittent application of force such as the use of a night splint only in post-operative Dupuytren's disease.

The use of composite joint motion, that is adding MCPJ, PIPJ and DIPJ range, is a potential limitation, however separate analysis by joint means multiple hypotheses tests and increases the risk of a Type I error. It is also widely acknowledged that correction of PIPJ contractures presents a greater challenge than in patients with MCPJ involvement only. The trial was not adequately powered for subgroup analyses and hence results of such secondary analyses need to be interpreted with caution. We did conduct one such subgroup analysis based on those patients who had surgery involving the PIPJ of the little finger (n = 109, 71% of total sample). No statistically significant differences were found between the groups on DASH or PIPJ flexion or extension. These results concur with the the primary analysis.

Our trial was pragmatic and its broad inclusion criteria, the fact that it was a multi-centre trial across 5 hospitals and involved 16 surgeons and around 26 therapists mean that generalisability is good. The baseline clinical and demographic characteristics of the whole cohort of 154 patients is typical of this patient group and although we were unable to collect data on non-consenting patients the fact that 73% of those invited consented, further supports the external validity of this trial. Furthermore we have no evidence of selection bias in the method by which surgeons screened patients for eligibility.

The interventions used in this pragmatic trial were consistent with current practice within the 5 trusts and we used practical criteria based on clinical reasoning to agree protocol deviations, although the exact thresholds for net loss of extension and therefore when to apply a splint were arbitrary and could be reconsidered.

Our results are generalisable to patients managed surgically by fasciectomy or dermofasciectomy, however caution is needed in extrapolating these findings to other surgical or less invasive procedures where the role of post-operative hand therapy including splinting remains largely unknown.

## Conclusions

Contrary to the widespread belief in the value of post-op night splinting for up to 6 months after fasciectomy or dermofasciectomy we found no evidence of its short or long-term effect. The policy of splinting all patients referred for hand therapy immediately after fasciectomy or dermofasciectomy needs to be reconsidered. This phase III trial provides evidence of a lack of effect of splinting when adhered to for at least 50% of nights in the first 3 months compared to hand therapy and splinting only when contractures occur. Patients receiving post-operative hand therapy by a specialised occupational therapist or physiotherapist had at least as good an outcome as those given an additional night splint. Only a small proportion (17%) of patients allocated to hand therapy went on to develop contractures of the PIPJ which exceeded the agreed threshold and needed a splint. Given the added expense of therapists' time, thermoplastic materials and the potential inconvenience to patients having to wear a device the policy of routine addition of night-time splinting after fasciectomy or dermofasciectomy should no longer be advocated but only where a loss of extension has occurred, particularly if the loss is rapid and early in the post-operative period.

## Competing interests

The authors declare that they have no competing interests.

## Authors' contributions

CJH, LS, AJC and DL all contributed to the trial concept and design, interpretation of data and drafting of the manuscript. LS undertook the statistical analysis. EB and SV conducted all data collection. CJH, LS, AJC and DL obtained funding. All authors revised the manuscript for important intellectual content and have read and approved the final version.

## Pre-publication history

The pre-publication history for this paper can be accessed here:

http://www.biomedcentral.com/1471-2474/12/136/prepub
